# Laboratory Validation of a Real-Time RT-PCR Assay for the Detection of Jamestown Canyon Virus

**DOI:** 10.3390/pathogens11050536

**Published:** 2022-05-03

**Authors:** Holly R. Hughes, Joan L. Kenney, Brandy J. Russell, Amy J. Lambert

**Affiliations:** Arboviral Diseases Branch, Division of Vector-Borne Diseases, Centers for Disease Control and Prevention, Fort Collins, CO 80521, USA; vwx1@cdc.gov (J.L.K.); bmk8@cdc.gov (B.J.R.); ahk7@cdc.gov (A.J.L.)

**Keywords:** Jamestown Canyon virus, La Crosse virus, real-time RT-PCR, diagnostic assay, surveillance

## Abstract

The neuroinvasive disease caused by Jamestown Canyon virus (JCV) infection is rare. However, increasing incidence and widespread occurrence of the infection make JCV a growing public health concern. Presently, clinical diagnosis is achieved through serological testing, and mosquito pool surveillance requires virus isolation and identification. A rapid molecular detection test, such as real-time RT-PCR, for diagnosis and surveillance of JCV has not been widely utilized. To enhance testing and surveillance, here, we describe the development and validation of a real-time RT-PCR test for the detection of JCV RNA. Three primer and probe sets were evaluated for analytical sensitivity and specificity. One probe set, JCV132FAM, was found to be the most sensitive test detecting 7.2 genomic equivalents/µL. While less sensitive, a second probe set JCV231cFAM was the most specific test with limited detection of Keystone virus at high RNA loads. Taken together, these data indicate both probe sets can be utilized for a primary sensitive screening assay and a secondary specific confirmatory assay. While both primer and probe sets detected high viral loads of Keystone virus, these assays did not detect any virus in the California encephalitis virus clade, including negative detection of the medically important La Crosse virus (LACV) and snowshoe hare virus (SSHV). The real-time RT-PCR assay described herein could be utilized in diagnosis and surveillance in regions with co-circulation of JCV and LACV or SSHV to inform public health action.

## 1. Introduction

Jamestown Canyon virus (JCV) is a mosquito-borne arbovirus in the genus *Orthobunyavirus*, with a genome consisting of three segments of negative-sense RNA. JCV was first isolated from a pool of *Culiseta inornata* mosquitoes from Jamestown Canyon, Colorado, in 1961 [1,2]. Since its initial identification, JCV is considered to be widespread throughout temperate North America with isolations from more than 26 different mosquito species, including some mosquito species that are also known vectors of La Crosse virus (LACV) [3]. While the vertebrate host of JCV is unknown, seroprevalence studies have found neutralizing antibodies in many domestic and wild mammals throughout North America and suggests white-tailed deer could be an amplification host [4,5,6].

JCV was originally serologically classified into the California serogroup, 1 of 18 serogroups in the genus *Orthobunyavirus* [7]. Through genomic evaluation, the California serogroup currently contains 11 species, including medically important *La Crosse orthobunyavirus*, *Snowshoe hare orthobunyavirus*, and *Jamestown canyon orthobunyavirus* [8]. These species include several virus strains known to cause human disease in North America and Europe: LACV, snowshoe hare virus, Khatanga virus, Inkoo virus (INKV), and JCV. The species *Jamestown canyon orthobunyavirus* also includes virus strains not known to cause human disease: Jerry Slough virus (JSV) and South River virus (SORV) [9].

JCV infection in humans is largely asymptomatic but can manifest as an acute febrile illness or neuroinvasive disease of meningitis or encephalitis [10]. Since its first description as a human pathogen in 1982 [11], JCV disease is considered rare with an average of 16 neuroinvasive diseases cases a year, although an increase in incidence has been described in recent years, likely associated with heightened awareness and testing detecting 75 cases in 2017 [10,12,13,14,15]. JCV infection is likely underrecognized in humans, and serosurveillance studies have suggested an antibody prevalence rate of 15 to 54% [6,16]. Diagnosis of JCV infection predominantly occurs through serological testing of JCV-specific immunoglobulin M and neutralizing antibodies [10]. Presently, diagnosis using molecular detection of JCV is limited due to unknown and likely low-level viremia. A recent study describing JCV clinical presentation failed to detect JCV RNA in cerebrospinal fluid (CSF) specimens as acute as three days post symptom onset [17]. Despite these limitations, molecular detection of JCV and other neuroinvasive arboviruses has become an important tool for diagnosis in immunocompromised individuals without detectable antibody responses [12,18,19,20]. Additionally, molecular detection of JCV in mosquito surveillance could be valuable to inform public health action. Herein, we describe the laboratory validation of a real-time RT-PCR assay to detect JCV RNA, including an evaluation of analytical and clinical specificity, to enhance diagnosis and surveillance.

## 2. Results

### 2.1. Evaluation of the JCV Real-Time RT-PCR Primers and Probes

**Figure 1:**
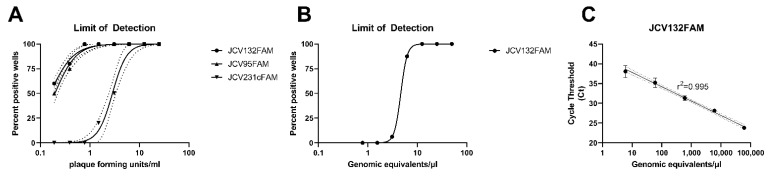
Limit of detection (LOD) and screening of Jamestown Canyon virus primers. (**A**) All three primer sets were screened for LOD against serially titrated virus stocks. The most sensitive primer and probe set was used for (**B**) analytical limit of detection determination and (**C**) determination of calibration curves on in vitro transcribed RNA. Regression lines indicate percent of positive detection at each dilution. The 95% confidence interval of each regression is indicated with dotted lines. The 95% limit of detection was interpolated for each regression. The Spearman correlation coefficient = r^2^.

The limit of detection for all small segment targeted primer sets (Table 1, Figure S1) was initially determined by evaluation of RNA extracts from serially titrated virus. The regression analysis determined the 95% LOD for the JCV132FAM probe set was 0.78 PFU/mL (95% CI 1.17–0.51 PFU/mL), the JCV95FAM probe set was 0.9 pfu/mL (95% CI 1.3–0.6 PFU/mL), while the JCV231cFAM probe set was the least sensitive, detecting 6.5 pfu/mL (95% CI 7.9–5.2 PFU/mL) (Figure 1A). Based on these results, the JCV132FAM probe set was selected for further evaluation. The analytical limit of detection, inter- and intra-assay precision for the JCV132FAM probe set was evaluated on serially titrated in vitro transcribed RNA copy controls. The 95% limit of detection of RNA copy controls was 7.19 copies/µL (95% CI 7.21–7.17) (Figure 1B). The intra-assay precision for replicates of serially diluted RNA copy controls ranged from 0.2 to 1.4 Ct standard deviations, with the greatest deviation for values near the limit of detection. The inter-assay coefficient of variation ranged from 0.6% to 3.7%, with the highest variation for the RNA copy load near the limit of detection. The dynamic range of the JCV132FAM primer set was determined by utilizing a standard curve of genomic equivalents (Figure 1C). Resulting Ct values correlated with the concentration of genomic equivalent copies (r^2^ = 0.995).

### 2.2. Analytical Specificity

The specificity of the JCV probe sets were determined by testing standardized RNA from 5 log_10_ PFU/mL of isolates of JCV species and additional California serogroup viruses (Table 2). All three probe sets detected all JCVs with Ct values that correlated to the limit of detection described above, and JCV132FAM consistently resulted in the lowest Ct values. Probe set JCV95FAM was the least specific and detected Serra do Navio virus (SDNV), Keystone virus (KEYV), and Melao virus (MELV). Probe set JCV231cFAM was the most specific and only detected KEYV RNA in addition to JCV species RNA, while probe set JCV132FAM had an intermediate specificity and detected KEYV and SDNV RNAs at high RNA levels. None of the three probe sets detected the RNA of viruses in the California encephalitis virus (CEV) clade, including CEV, LACV, and SSHV. The ability of the primers to detect RNA of JCV viruses from multiple lineages was investigated. RNA from 11 additional JCVs from lineages A, B1, and B2 [21] were all detected by all primers and probes.

### 2.3. Clinical Sensitivity

Three specimens from a previously confirmed JCV neuroinvasive case were tested by the probe set, JCV132FAM. JCV RNA from FFPE cortex, cerebellum, and serum was successfully detected with Ct values of 24.5, 37.3, and 35.7, respectively. Archived specimens from six additional cases without evidence of JCV infection were found to be negative by probe JCV132FAM, suggesting clinical specificity of the real-time RT-PCR assay.

### 2.4. Mosquito Pool Testing

Contrived JCV spiked mosquito pools of 50 *Culex pipiens* were utilized for preliminary evaluation of JCV primers sets in mosquito pool surveillance (Table 3). All three primer sets detected JCV RNA when extracted from spiked mosquito pools.

## 3. Discussion

Jamestown Canyon virus is an endemic, emerging neuroinvasive arbovirus of public health concern [22] belonging to the California serogroup [7]. Herein, we describe the development and validation of a fluorogenic-probe-based real-time RT-PCR test for the successful detection of RNA from JCV strains in clinical specimens, isolates, and preliminary evaluation of mosquito pools. Of the three probe sets investigated in this study, probe set JCV132FAM was the most sensitive detecting 7.2 RNA copies/µL, while JCV231cFAM was most specific detecting only high titer KEYV in addition to JCV. Taken together, these data indicate that the probe set JCV132FAM could be utilized as a primary screening test, and JCV231cFAM could be employed as a secondary or confirmatory set if higher specificity is required through clinical diagnostic testing.

The California serogroup can be phylogenetically divided into three clades, CEV, MELV, and TVTV [23,24], with the medically important LACV in the CEV clade and JCV in the MELV clade. While the California serogroup viruses form distinct clades, the sequences of the S segment are especially conserved across the group [23,25]. This conserved S segment sequence underlies the detection of all virus strains classified as *Jamestown canyon orthobunyavirus* (e.g., JCV, INKV, JSV, SORV). This conservation and detection are especially highlighted by the low Ct values of JCV primer sets with SORV, which binds with 100% nucleotide agreement. Alternatively, lower Ct values seen with some JCV species could be due to the detection of defective viral genomes elicited through passage in cell culture [26]. Nonetheless, the detection of a broad range of JCV species using standardized, infectious virus was successful. Although, probe set JCV132FAM and JCV231cFAM also detected KEYV and SDNV, the high viral loads greater than 4 log_10_ PFU/mL suggests these probe sets would be clinically specific given the low level or undetectable viremia of KEYV [27,28] and lack of recognized human disease by SDNV [29]. Recently, Kinsella et al. developed a real-time RT-PCR sybrgreen-based assay to detect JCV RNA in clinical cases and vectors [17]. The assay described in this study has demonstrated similar specificity with an in silico analysis, suggesting the previously described assay detects all strains of JCV species, although specificity data were not reported [17]. Differences in the detection methodology and validation, reliant on DNA standards in the previous assay, makes the comparison of sensitivity to the test described here unreliable.

Jamestown Canyon virus is likely an underreported arboviral disease in the United States [10]; however, increasing incidence has been observed with 45 clinical cases reported in 2019 [13]. Presently, serological testing is the gold standard for diagnosis of JCV [10,17] due to the low viral loads and short-lived viremia [17,30]. Although serological diagnosis will remain the gold standard for JCV and neuroinvasive arboviruses, molecular detection methods have been instrumental tools in detecting arboviral infections in immunocompromised individuals [12,19]. While the utility of molecular clinical diagnosis for JCV remains limited, the real-time RT-PCR assay described herein may be a valuable tool for enhancing JCV surveillance in mosquito vectors. Preliminary evaluation of the JCV primers demonstrated successful detection of JCV in mosquito pools; however, a more detailed evaluation of mosquito pool testing is warranted. JCV has been identified in >26 different mosquito species [3], including species that are known or potential vectors of LACV [31]. Given a lack of cross-amplification with LACV RNA, this assay could be utilized in surveillance in the Great Lakes region and Midwestern states, where JCV and LACV are known to co-circulate, informing public health action for these important arboviruses.

## 4. Materials and Methods

Unless otherwise noted, all procedures were followed according to the manufacturer’s protocols.

### 4.1. Primers and Probes

A total of 109 JCV complete S segment sequences in addition to INKV, JSV, and SORV, were downloaded from GenBank and aligned using the Clustal W function of MEGA 7 software. The prototype JCV strain 61V2235 (NC_043558) S segment sequence was submitted to the Primer Quest software (IDT, Coralville, IA, USA) for primer and probe design. Alignments were evaluated with potential primer and probe sets for specificity across JCV strains, and three primer and probe sets were selected and synthesized (IDT) (Table 1). All probes contained 5′FAM and 3′IowaBlack quencher.

### 4.2. Viruses and RNA

Viruses used for sensitivity and specificity in the study were provided by the Arboviral Diseases Branch, Division of Vector-Borne Diseases, Centers for Disease Control and Prevention’s (CDC) Arbovirus Reference Collection (ARC). Isolate details are described in Table 2. RNA was extracted from frozen stocks using the QIAmp Viral RNA mini kit (Qiagen, Germantown, MD, USA).

### 4.3. Standardization of JCV Real-Time RT-PCR:

The JCV real-time RT-PCR was standardized using the Quantitect probe RT-PCR kit (Qiagen) with 1 µM each primer and 0.2 µM FAM labeled probe with the following cycling conditions: 50 °C for 30 min; 95 °C for 15 min; 45 cycles of 94 °C for 15 s; and 60 °C for 1 min on the CFX96 Real-time PCR detection system (Bio-Rad, Hercules, CA, USA). Ten microliters of RNA were used in a final reaction volume of twenty-five microliters. Each RNA sample was tested with a minimum of two technical replicates, and each assay included at least two non-template negative controls, negative extraction controls, and JCV RNA-positive controls. Cycle threshold (Ct) values greater than 38.0 were considered negative.

### 4.4. Standard Preparation for JCV Real-Time RT-PCR Assay

JCV 61V2235 S segment genomic RNA from cell culture supernatants was reverse transcribed and amplified using the Qiagen One-step RT-PCR kit as previously described [32]. Amplicons were checked for appropriate 900 bp size on 1.2% E-gel (Thermo Fisher, Waltham, MD, USA). Unpurified amplification products were cloned into the TOPO TA dual promoter kit (Thermo Fisher). White-blue screening of transformed colonies was completed on imMedia AmpBlue plates (Thermo Fisher), and plasmid DNA was extracted using Miniprep kit (Qiagen). The JCV S segment insert was verified using the sequencing primers provided in the TOPO TA kits, and BigDye Terminator sequencing (Thermo Fisher) was performed using the ABI 3130 instrument (Thermo Fisher).

RNA copy controls were in vitro transcribed after linearization of the plasmid with 1 U KpnI-HF (New England BioLabs, Ipswich, MA, USA) for 1 hr at 37 °C. Enzymatic reactions were cleaned using the QiaQuick PCR clean up kit (Qiagen). RNA was synthesized using 500 ng of linearized plasmid in the mMessage mMachine T7 Ultra kit (Ambion, Austin, TX, USA) and incubated at 37 °C for 5 hr, followed by Turbo DNase treatment. Transcribed RNAs were purified using the RNeasy kit (Qiagen), RNA concentration determined using the Nanodrop One spectrophotometer (Thermo Fisher) and analyzed with the 2200 Tapestation Bioanalyzer (Agilent). RNA copy number was estimated using the following equation: number of copies= [RNA concentration (ng) * 6.022 × 10^23^]/ [length (bp) * 1 ×10^9^ * 340] (www.scienceprimer.com, accessed on 15 October 2019). Serial dilutions were made in molecular grade water to generate a standard curve ranging in 1 × 10^5^ copies/reaction to 1 copy/reaction.

### 4.5. Determination of Analytical Performance

To determine the limit of detection (LOD) of the JCV assay, 5 replicates of serial dilutions of JCV 61V2235 cell culture supernatant in the range of 50 plaque-forming units (PFU)/mL to 0.1 PFU/mL were tested in 2 independent assays. Likewise, in vitro transcribed RNA copy controls in the range of 250 copies/reaction to 1 copy/reaction were tested in 8 replicates in 2 independent assays. The percent positive (detected) wells were plotted on a non-linear regression using a Sigmoidal, 4-parameter curve. Constrains were set to 100 and 0% to reflect boundaries of data. The concentration with 95% detection was interpolated using GraphPad Prism 9 (GraphPad Software Inc, San Diego, CA, USA). Standard deviation of the intra-assay Ct and coefficient of variation (CV) of the inter-assay variance was also calculated in GraphPad Prism 9.

Standard curves for JCV132FAM were generated with 10-fold dilutions of RNA genomic equivalents in water. RNA in the ranges of 6 × 10^4^ to 6 × 10^0^ genomic equivalents/ µL were tested in quadruplicate as described above. Resulting Ct values were plotted, and the Spearman coefficient of correlation (r^2^) was calculated in GraphPad Prism 9.

Specificity of the assay was determined by testing for potential cross-reactivity with 10 µL of RNA extracted from 5 log_10_ PFU/mL of virus cell culture supernatants of different California serogroup isolates from all lineages (Table 2), in 2 independent assays.

### 4.6. Clinical Analysis

Archived specimens from a previously confirmed fatal case of JCV neuroinvasive disease were evaluated [12]. RNA extracted from formalin-fixed paraffin-embedded (FFPE) tissues was previously received from the CDC Infectious Diseases Pathology Branch, and RNA was extracted from 140 µL of serum using the QIAmp Viral RNA mini kit (Qiagen). Residual archived CSF samples from JCV negative cases were used following using CDC’s Institutional review board protocol 6773.

### 4.7. Mosquito Pool Testing

Mosquito pools from in-house bred *Culex pipiens* were used to determine suitability of the JCV primer sets in mosquito surveillance. Serial dilution of JCV 61V2235 were spiked into pools of 50 *Cx. Pipiens* in the range of 6 log_10_ PFU/mL to 4 log_10_ PFU/mL. Pools were process as previously described [33]. Briefly, pools of 50 adult *Cx. Pipiens* were homogenized with 1 mL BA-1 media in a Mixer Mill (Qiagen), clarified by centrifugation, RNA was extracted using the MagMax Viral Pathogen Nucleic Acid kit (Thermo Fisher) and eluted in 100µL. Ten microliters of eluate from each pool and a negative pool were tested in duplicate with each JCV primer set with the conditions described above.

## Figures and Tables

**Table 1:** Primer and probes evaluated in this study.

Primer Name ^1^	Sequence 5′–3′	Limit of Detection (95% Confidence)	
		Plaque Forming Units/mL	Genomic Equivalents/µL
JCV174	CAGTCTGTCAGCCGTTAGGA	6.5 (7.9–5.2)	ND ^2^
JCV269c	AATTTCCACCTGCCACTCTC		
JCV231cFAM	TCCGCTCCGGTTTACGAGCG		
JCV102	ATCCACAGGTGCAAATGGA	0.8 (1.17–0.51)	7.19 (7.21–7.17)
JCV201c	GAAGAAGATCCTAACGGCTGMC		
JCV132FAM	TGCAGGGTTTGTGGCATTTATGGC		
JCV58	GCATACTTGGTTGATATGGGAGA	0.9 (1.2–0.5)	ND
JCV155c	GCCATAAATGCCACAAACCC		
JCV95FAM	ATGTCGCATCCACAGGTGCAAATG		

^1^ Genomic position based on small segment GenBank accession number U127961. ^2^ Not Determined (ND).

**Table 2:** Specificity of primers and probes on California serogroup viruses.

Virus Species	Virus Name	Isolate	Location	Year	Average Cycle Threshold (Ct) ^1^		
					JCV 132FAM	JCV 95FAM	JCV 231cFAM
*Jamestown canyon orthobunyavirus*	Jamestown Canyon	61V2235	Colorado, USA	1961	18.2	20.7	23.1
		L36708 (lineage A)	Connecticut, USA	1966	18.1	18.7	23.1
		MN256-260	Manitoba, Canada	1979	17.2	17.4	18.6
		1262-98 (lineage B1)	Connecticut, USA	1998	20.8	23.3	26.3
		515-99 (lineage A)	Connecticut, USA	1999	19.2	20.9	21.4
		4473-00 (lineage B2)	Connecticut, USA	2000	21.2	22.5	26.4
		1425-02 (lineage B1)	Connecticut, USA	2002	21.1	22.3	25.1
		4148-03 (lineage B2)	Connecticut, USA	2003	20.2	24.0	27.1
		11497-03 (lineage A)	Connecticut, USA	2003	19.4	22.1	22.7
		1441-04 (lineage B1)	Connecticut, USA	2004	20.1	21.2	24.1
		3836-05 (lineage A)	Connecticut, USA	2005	19.0	20.7	21.6
		NM5-4BU	New Mexico, USA	1977	21.0	23.5	25.0
	Jerry Slough	BFS4474	California, USA	1963	21.0	23.3	25.8
	Inkoo	KN3641	Jukon, Finland	1964	18.1	21.4	26.2
	South River	NJO-94F	New Jersey, USA	1960	13.4	15.2	17.8
*Keystone orthobunyavirus*	Keystone	B64-5587.05	Florida, USA	1964	23.8	35.5	27.2
*Serra do Navio orthobunyavirus*	Serra do Navio	BeAr 103645	Amapa, Brazil	1966	34.5	29.0	Negative
*Melao orthobunyavirus*	Melao	TRVL 9375	Trinidad	1955	Negative	37.03	Negative
*California encephalitis orthobunyavirus*	California encephalitis	BFS 283	California, USA	1943	Negative	Negative	Negative
*La Crosse orthobunyavirus*	La Crosse	Original (Human/1960)	Wisconsin, USA	1960	Negative	Negative	Negative
*snowshoe hare orthobunyavirus*	snowshoe hare	Original (Montana 1959)	Montana, USA	1959	Negative	Negative	Negative

^1^ Average Cycle threshold (Ct) of two independent experiments tested on RNA extracted from 5 log_10_ PFU/mL of virus.

**Table 3:** Detection of Jamestown canyon virus from spiked mosquito pools.

	Average Cycle threshold (Ct) ^2^		
Mosquito Pool ^1^	JCV132FAM	JCV95FAM	JCV231cFAM
6 log_10_ PFU/mL	18.4	19.2	19.9
5 log_10_ PFU/mL	22.5	23.0	24.1
4 log_10_ PFU/mL	24.8	25.9	26.2
Negative pool	Negative	Negative	Negative

^1^ Mosquito pools spiked with JCV at specified plaque forming units per mL (PFU/mL) or negative control pool; ^2^ Average Cycle threshold (Ct) of two independent experiments

## Data Availability

All data applicable to this study are presented herein.

## Supplementary Materials

The following supporting information can be downloaded at https://www.mdpi.com/article/10.3390/pathogens11050536/s1, Figure S1: Map of Jamestown Canyon virus small segment and real-time RT-PCR primer sets. Schematic representation of the location of each primer set.

## References

[1] Pastula D.M., Smith D.E., Beckham J.D., Tyler K.L. (2016). Four emerging arboviral diseases in North America: Jamestown Canyon, Powassan, chikungunya, and Zika virus diseases. J. Neurovirol..

[2] Campbell G.L., Eldridge B.F., Reeves W.C., Hardy J.L. (1991). Isolation of Jamestown Canyon virus from boreal Aedes mosquitoes from the Sierra Nevada of California. Am. J. Trop. Med. Hyg..

[3] Andreadis T.G., Anderson J.F., Armstrong P.M., Main A.J. (2008). Isolations of Jamestown Canyon virus (Bunyaviridae: Orthobunyavirus) from field-collected mosquitoes (Diptera: Culicidae) in Connecticut, USA: A ten-year analysis, 1997–2006. Vector Borne Zoonotic Dis..

[4] Boromisa R.D., Grimstad P.R. (1987). Seroconversion rates to Jamestown Canyon virus among six populations of white-tailed deer (*Odocoileus virginianus*) in Indiana. J. Wildl. Dis..

[5] Fulhorst C.F., Hardy J.L., Eldridge B.F., Chiles R.E., Reeves W.C. (1996). Ecology of Jamestown Canyon virus (Bunyaviridae: California serogroup) in coastal California. Am. J. Trop. Med. Hyg..

[6] Zarnke R.L., Calisher C.H., Kerschner J. (1983). Serologic evidence of arbovirus infections in humans and wild animals in Alaska. J. Wildl. Dis..

[7] Calisher C.H. (1996). The Bunyaviridae.

[8] Hughes H.R., Adkins S., Alkhovskiy S., Beer M., Blair C., Calisher C.H., Drebot M., Lambert A.J., de Souza W.M., Marklewitz M. (2020). ICTV Virus Taxonomy Profile: Peribunyaviridae. J. Gen. Virol..

[9] Rust R.S., Thompson W.H., Matthews C.G., Beaty B.J., Chun R.W. (1999). La Crosse and other forms of California encephalitis. J. Child. Neurol..

[10] Pastula D.M., Hoang Johnson D.K., White J.L., Dupuis A.P., Fischer M., Staples J.E. (2015). Jamestown Canyon Virus Disease in the United States-2000–2013. Am. J. Trop. Med. Hyg..

[11] Grimstad P.R., Shabino C.L., Calisher C.H., Waldman R.J. (1982). A case of encephalitis in a human associated with a serologic rise to Jamestown Canyon virus. Am. J. Trop. Med. Hyg..

[12] Solomon I.H., Ganesh V.S., Yu G., Deng X.D., Wilson M.R., Miller S., Milligan T.A., Mukerji S.S., Mathewson A., Linxweiler J. (2021). Fatal Case of Chronic Jamestown Canyon Virus Encephalitis Diagnosed by Metagenomic Sequencing in Patient Receiving Rituximab. Emerg. Infect. Dis..

[13] Vahey G.M., Mathis S., Martin S.W., Gould C.V., Staples J.E., Lindsey N.P. (2021). West Nile Virus and Other Domestic Nationally Notifiable Arboviral Diseases—United States, 2019. MMWR Morb. Mortal. Wkly Rep..

[14] Matkovic E., Hoang Johnson D.K., Staples J.E., Mora-Pinzon M.C., Elbadawi L.I., Osborn R.A., Warshauer D.M., Wegner M.V., Davis J.P. (2019). Enhanced Arboviral Surveillance to Increase Detection of Jamestown Canyon Virus Infections, Wisconsin, 2011–2016. Am. J. Trop. Med. Hyg..

[15] Curren E.J., Lehman J., Kolsin J., Walker W.L., Martin S.W., Staples J.E., Hills S.L., Gould C.V., Rabe I.B., Fischer M. (2018). West Nile Virus and Other Nationally Notifiable Arboviral Diseases—United States, 2017. MMWR Morb. Mortal. Wkly Rep..

[16] Mayo D., Karabatsos N., Scarano F.J., Brennan T., Buck D., Fiorentino T., Mennone J., Tran S. (2001). Jamestown Canyon virus: Seroprevalence in Connecticut. Emerg. Infect. Dis..

[17] Kinsella C.M., Paras M.L., Smole S., Mehta S., Ganesh V., Chen L.H., McQuillen D.P., Shah R., Chan J., Osborne M. (2020). Jamestown Canyon virus in Massachusetts: Clinical case series and vector screening. Emerg. Microbes Infect..

[18] Solomon I.H., Spera K.M., Ryan S.L., Helgager J., Andrici J., Zaki S.R., Vaitkevicius H., Leon K.E., Wilson M.R., DeRisi J.L. (2018). Fatal Powassan Encephalitis (Deer Tick Virus, Lineage II) in a Patient with Fever and Orchitis Receiving Rituximab. JAMA Neurol..

[19] Hughes H.R., Velez J.O., Davis E.H., Laven J., Gould C.V., Panella A.J., Lambert A.J., Staples J.E., Brault A.C. (2021). Fatal Human Infection with Evidence of Intrahost Variation of Eastern Equine Encephalitis Virus, Alabama, USA, 2019. Emerg. Infect. Dis..

[20] Solomon I.H., Ciarlini P., Santagata S., Ahmed A.A., De Girolami U., Prasad S., Mukerji S.S. (2017). Fatal Eastern Equine Encephalitis in a Patient on Maintenance Rituximab: A Case Report. Open Forum Infect. Dis..

[21] Armstrong P.M., Andreadis T.G. (2007). Genetic relationships of Jamestown Canyon virus strains infecting mosquitoes collected in Connecticut. Am. J. Trop. Med. Hyg..

[22] Gill C.M., Beckham J.D., Piquet A.L., Tyler K.L., Pastula D.M. (2019). Five Emerging Neuroinvasive Arboviral Diseases: Cache Valley, Eastern Equine Encephalitis, Jamestown Canyon, Powassan, and Usutu. Semin. Neurol..

[23] Bowen M.D., Jackson A.O., Bruns T.D., Hacker D.L., Hardy J.L. (1995). Determination and comparative analysis of the small RNA genomic sequences of California encephalitis, Jamestown Canyon, Jerry Slough, Melao, Keystone and Trivittatus viruses (Bunyaviridae, genus Bunyavirus, California serogroup). J. Gen. Virol..

[24] Campbell W.P., Huang C. (1999). Sequence comparisons of medium RNA segment among 15 California serogroup viruses. Virus Res..

[25] Hughes H.R., Lanciotti R.S., Blair C.D., Lambert A.J. (2017). Full genomic characterization of California serogroup viruses, genus Orthobunyavirus, family Peribunyaviridae including phylogenetic relationships. Virology.

[26] Levin J.G., Ramseur J.M., Grimley P.M. (1973). Host effect on arbovirus replication: Appearance of defective interfering particles in murine cells. J. Virol..

[27] Lednicky J.A., White S.K., Stephenson C.J., Cherabuddi K., Loeb J.C., Moussatche N., Lednicky A., Morris J.G. (2019). Keystone Virus Isolated from a Florida Teenager with Rash and Subjective Fever: Another Endemic Arbovirus in the Southeastern United States?. Clin. Infect. Dis..

[28] Watts D.M., Bailey C.L., Roberts N.T., RF T.A., Dalrymple J.M., Clark G.C. (1988). Maintenance and transmission of Keystone virus by Aedes atlanticus (Diptera: Culicidae) and the gray squirrel in the Pocomoke Cypress Swamp, Maryland. J. Med. Entomol..

[29] Evans A.B., Peterson K.E. (2019). Throw out the Map: Neuropathogenesis of the Globally Expanding California Serogroup of Orthobunyaviruses. Viruses.

[30] Bennett R.S., Nelson J.T., Gresko A.K., Murphy B.R., Whitehead S.S. (2011). The full genome sequence of three strains of Jamestown Canyon virus and their pathogenesis in mice or monkeys. Virol. J..

[31] Harris M.C., Yang F., Jackson D.M., Dotseth E.J., Paulson S.L., Hawley D.M. (2015). La Crosse Virus Field Detection and Vector Competence of Culex Mosquitoes. Am. J. Trop. Med. Hyg..

[32] Lambert A.J., Lanciotti R.S. (2008). Molecular characterization of medically important viruses of the genus Orthobunyavirus. J. Gen. Virol..

[33] Savage H.M., Ledermann J.P., Yug L., Burkhalter K.L., Marfel M., Hancock W.T. (2015). Incrimination of Aedes (Stegomyia) hensilli Farner as an epidemic vector of Chikungunya virus on Yap Island, Federated States of Micronesia, 2013. Am. J. Trop. Med. Hyg..

