# Supplementary material for: Laboratory Validation of a Real-Time RT-PCR Assay for the Detection of Jamestown Canyon Virus
Source: Pathogens. 2022 May 3;11(5):536. doi: 10.3390/pathogens11050536 (PMC9146205; doi:10.3390/pathogens11050536)
Supplement: Supplementary file 1 [file pathogens-11-00536-s001.zip › pathogens-1675498-supplementary.pdf]

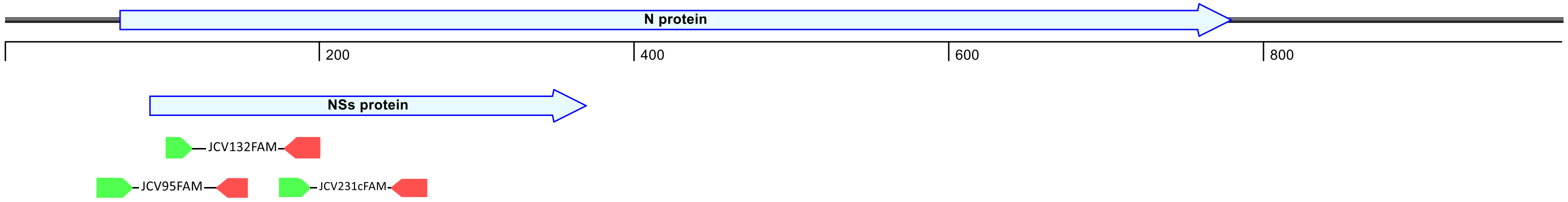

Figure S1. Map of Jamestown Canyon virus small segment and real-time RT-PCR primer sets. Schematic representation of the location of each primer set. Green arrows indicate forward primers, red arrows indicate reverse primers. N=nucleoprotein, NSs= non-structural small protein.
